# Medicinal Compositions of Long Stephania Herb Exhibit Antilung Cancer Effects

**DOI:** 10.1155/adpp/8058846

**Published:** 2026-10-28

**Authors:** Xiaobing Chen, Luying Zhang, Wanying Zhang, Xueqin Wu, Kun Liu, Yuli Zhou, Yongqing Chen, Mingyue Zhu, Bo Lin, Mengsen Li

**Affiliations:** ^1^ Key Laboratory of Tropical Translational Medicine Ministry of Education, and Hainan Provincial Key Laboratory of Carcinogenesis and Intervention Hainan Medical University Haikou 571199 Hainan China hainmc.edu.cn; ^2^ Department of Respiratory and Critical Care Medicine Hainan General Hospital Hainan Affiliated Hospital of Hainan Medical University Haikou 570311 Hainan China hyfyuan.com; ^3^ Department of Medical Oncology Second Affiliated Hospital Hainan Medical University Haikou 570216 Hainan China hainmc.edu.cn

**Keywords:** apoptosis, cancer treatment, long-stephania herb, medicinal composition, nonsmall cell lung cancer

## Abstract

Lung cancer is one of the most common malignancies worldwide, and due to its high mortality rate and resistance to treatment, there is an urgent need to develop effective treatments with low toxicity. Long stephania herb (LSH) is a tropical herb used clinically for the treatment of inflammation and analgesia, but its antitumor effects are unknown. In this study, UHPLC–OE–MS was used to detect the chemical components of LSH; the cytotoxic effects of these chemical components on normal lung cells and non‐small cell lung cancer (NSCLC) cell lines were observed in vitro cell experiments, and the inhibitory effects of LSH on the growth of NSCLC were detected in animal tumor models. The possible molecular targets of LSH for NSCLC were predicted by network pharmacology and molecular docking. The results showed that LSH has more than a thousand chemical components; at the microgram level per milliliter, it can effectively inhibit the proliferation, migration, and invasion of lung cancer A549 cells and promote apoptosis in these cells. It also can effectively inhibit the growth of tumors *in vivo* and has no significant toxic effects on normal lungs, liver, heart, and kidneys. Analysis showed that 20 pharmacodynamic components of LSH may target EGFR, PIK3CA, PIK3R1, AKT1, HSP90AA1, SRC, MAPK1, MAPK3, STAT3, JUN, and CASP3. Inhibition of the PI3K/AKT signaling pathway and proteoglycans in cancer and other signaling pathways plays an anti‐NSCLC role. In conclusion, this study is the first to report the efficacy of LSH in the treatment of NSCLC, and it is a very valuable tropical medicinal plant for the treatment of lung cancer.

## 1. Introduction

Lung cancer is the most common cancer in the world; non‐small cell lung cancer (NSCLC) accounts for about 85% of all lung cancer cases, according to the World Health Organization Cancer Statistics 2024 [1]. Worldwide, lung cancer has the highest number of deaths among various cancers, almost 2.5 times the number of deaths from colorectal cancer. With the in‐depth research of molecular targeted drugs, resulting in significant improvement in the treatment of lung cancer, the 5‐year survival rate of lung cancer has increased to 25%. There are often reported cases of chemotherapy failure due to serious adverse reactions, drug resistance, and disease progression [2]. Therefore, there is an urgent need to find safe and effective drugs to treat lung cancer.

Traditional Chinese medicine (TCM) has been treating diseases for thousands of years, and TCM treatments are widely accepted because they have fewer side effects than many commonly used chemotherapy drugs. TCM has a complex composition, has a variety of natural compounds that can be used for cancer prevention and as cancer chemotherapy drugs, and usually has multiple therapeutic targets, such as Banxia Xiexin decoction (BXD) for colorectal cancer [3]. Long stephania herb (LSH) belongs to the family Menispermaceae, Stephania, Latin name Stephania longa Lour, English name LSH. TCM doctors usually use it to treat patients suffering from diarrhea, edema, jaundice, and sore and poisonous snake bites. Chinese literature has reported a variety of effects, such as anti‐inflammatory and analgesic effects. The chloroform extract of LSH can inhibit the hyperplasia of the prostate in mice, and the ethanol extract of LSH can treat chronic suppurative otitis media. The non‐alkaloid extract of LSH has an obvious antibacterial effect on various bacteria such as *Staphylococcus aureus* and *Escherichia coli*. The methanol extracts from different parts of LSH, such as roots, stems, and leaves, can inhibit the activity of plant pathogenic fungi. LSH also has a diuretic sedative effect; LSH ethanol extract and non‐alkaloids have a diuretic effect on rats. In addition, its alkaloids have a sedative effect on mice. However, it is still unclear whether LSH has a preventive and therapeutic effect on various cancers, especially since no studies have reported the therapeutic effect of LSH on NSCLC. Network pharmacology can clarify the active ingredients and potential targets of drug action, as well as the interaction between active ingredients and multiple targets [4], which is suitable for the study of multitarget TCM drugs [5]. In this study, network pharmacology was used to predict the therapeutic effect of LSH on NSCLC, and the binding ability of LSH‐effective compounds and targets was predicted by molecular docking detection. This study found that the chemical composition of LSH has a significant therapeutic effect on NSCLC at the microgram level, while the toxicity to normal lung cells and other organs is very low. This study uses network pharmacology analysis, along with cellular and tumor model mouse analysis, to investigate the antitumor effects of LSH on lung cancer cells and explores the impact of LSH on the expression of apoptosis‐related factors within lung cancer cells at the molecular level. This study found a new effective herb for anti‐NSCLC.

## 2. Materials and Methods

### 2.1. Preparation of Medicinal Liquid Extracted From LSH

LSH was collected from Lingao County, Hainan Province, China, and 15 g of dried leaves of LSH were washed with pure water to remove all trace particles and pollutants such as dust. Place in a stainless steel electric boiling pot, add 1000 mL pure water, and soak for 1 h [6]. After boiling, continue to boil for 40 min, filter through a 70‐μm cell filter, and filter out the liquid medicine. The residue was then added to 800 mL of pure water, boiled, and filtered in the same method. This process was repeated twice for a total of 3 times, and the experimental procedure was based on Reference [7]. Pour all the liquid medicine into the stainless steel plate, put it into a vacuum drying oven at 70°C, and dry for 48 h under pressure to obtain LSH water to extract the liquid medicine into a solid paste. The liquid was weighed and dissolved in phosphate buffer saline (PBS) to form a high‐concentration liquid. The liquid was absorbed by a 5 mL disposable injector and filtered by a 0.22 μm sterile polyethersulfone (PES) membrane disposable needle filter. The sterile LSH water extract liquid was obtained and stored at 4°C.

### 2.2. Network Pharmacological Analysis

#### 2.2.1. Ultra High‐Performance Liquid Chromatography–Mass Spectrometry (UHPLC–OE–MS) Detection of LSH Components

Studies on the composition of LSH are limited, and there are few reports in the literature. The LSH water‐extracted medicinal liquid dry paste was stored on dry ice and sent to Biotree Technology Co., Ltd. (Shanghai, China) for ingredient testing. The active ingredient of LSH was analyzed by the UHPLC–OE–MS detection system. The target compounds were chromatographically separated using a Phenomenkinetexcel 18 (2.1 mm × 100 mm, 2.6 μm) liquid chromatograph using the Vanquish (ThermoFisher Scientific) ultra‐high performance liquid chromatograph. Phase A was aqueous, containing 0.01% acetic acid; phase B was isopropyl alcohol:acetonitrile (1:1, v/v). Sample tray temperature: 4°C, sample volume: 2 μL. The OrbitrapExploris120 mass spectrometer is capable of primary and secondary mass spectrometry data acquisition under the control of the control software (Xcalibur, Version 4.4, Thermo). Detailed parameters can be found in Biotree_report. This untargeted metabolomics is tested in both positive ion mode and negative ion mode.

Oral bioavailability (OB) stands for bioavailability, which refers to the rate and degree of drug absorption into human body circulation, reflecting the percentage of the oral dose of drug given into human body circulation. Drug‐like properties (DL) referring to the similarity of a compound to a known drug. A drug‐like compound is not a drug but has the potential to become a drug. According to the literature [8–10], the active ingredients were selected, and the OB value ≥ 30%, DL value ≥ 0.18, and MS2 score value were higher to screen the active ingredients [11].

#### 2.2.2. Prediction of LSH‐Related Targets and Collection of Targeted Molecules Related to NSCLC

SMILES was obtained by entering the PubChem IDs of the 20 selected active ingredients into the PubChem database (https://pubchem.ncBI.nlm.nih.gov/). SMILES will compound input the SwissTargetPrediction database (https://www.swisstargetprediction.ch/) to predict compound treatment targets, *Homo sapiens* species selection, and selection probability > 0 therapeutic targets [12]. The GeneCards (https://previous.genecards.org) database was used to search for targeted molecules related to NSCLC. In order to obtain targets with high reliability, targets with scores > 25 points were selected. Meanwhile, the OMIM (https://www.omim.org) database was selected to search for targeted molecules related to NSCLC, and the targets obtained from the GeneCards database and the OMIM database were integrated. Venny 2.1.0, an online tool, was used to draw a Venn diagram to screen overlapping targets between LSH and NSCLC, which are potential targeted molecules for LSH treatment of NSCLC [13].

#### 2.2.3. Construction of Protein–Protein Interaction (PPI) Networks and Screening of Core Targets

We used the STRING12.0 database (https://cn.string-db.org/) for analysis of protein interactions. Potential targets of LSH in the treatment of NSCLC were entered into the STRING database. Multiple proteins were selected, and the species *Homo sapiens* was selected. The “minimum required interaction score” was set to “high confidence > 0.7,” which ensured our analysis of the active target proteins corresponding to potential targets [14]. Import the results generated by STRING into the visual analysis application software Cytoscape software (Versions 3.7.2 and 3.9.1) to generate a PPI network diagram. The network analysis tool in the software is used to conduct topology analysis according to degree and combined score, and the topological characteristics of the nodes and edges of the PPI network can be calculated, including the intermediate degree, proximity degree, and degree value, and a potential target PPI network diagram for visual optimization can be created. Cytoscape software (Versions 3.7.2 and 3.9.1) was used to select core targets. The plug‐in CentiScape2.2 and the parameters betweenness, closeness, and degree were selected as screening indicators. Nodes corresponding to targets that meet the following conditions are selected as the core targets for LSH treatment of NSCLC. (1) Betweenness > threshold; (2) closeness > threshold; (3) degree > threshold. A network analysis tool was used to analyze the core target topology, and a PPI network diagram of the core target was created.

#### 2.2.4. Gene Ontology (GO) Analysis and Kyoto Encyclopedia of Genes and Genomes (KEGG) Pathway Enrichment Analysis of Overlapping Targets

GO analysis and KEGG pathway enrichment analysis for LSH in NSCLC were performed using the Metscape database (https://metascape.org/). The overlapping targets were uploaded to the Metscape database, the species was selected as *H. sapens*, and the parameters were set to MinOverlap = 3, *p* value cutoff = 0.01, and MinEnrichment = 1.5 [15]. GO analysis includes biological processes (BPs), cellular components (CCs), and molecular functions (MFs). After completing the analysis and obtaining the relevant data, sorted according to the log *p* value, and then selecting the *p* value that is the smallest before 15 GO results and, according to low *p* values, in KEGG pathways associated with NSCLC and 20 top KEGG pathways into the bioinformatics cartography website (https://www.bioinformatics.com.cn/). The classification histogram and bubble map were drawn, respectively. To effectively interpret and present the results of GO and KEGG analysis [16].

#### 2.2.5. LSH‐Active Component–Core Target–Signaling Pathway–NSCLC Network Construction

The 20 active components of LSH, the core targets of LSH in the treatment of NSCLC, and the data of 20 pathways in the bubble map of KEGG enrichment analysis were imported into Cytoscape 3.9.0 to map the LSH‐active components—core target—KEGG pathway—NSCLC network.

#### 2.2.6. Molecular Docking of Key Bioactive Ingredients With Core Targets

The DiscoveryStudio2022 software was used for computer virtual molecular docking to study the interaction between LSH’s key active components, namely ligands, and its key core target, namely receptor protein molecules, and predict the affinity. From the PubChem database (https://pubchem.ncbi.nlm.nih.gov/), download the key ingredient in SDF format 3D structure. 3D structures of key core targets (X‐ray diffraction) are downloaded in PDB format from the RCSBPDB database (https://www.rcsb.org). CDOCKER is a semiflexible docking program based on CHARMm, which uses high‐temperature dynamics to search the flexible conformation space of ligand molecules and uses the simulated annealing method to optimize each conformation at the receptor active site so as to make docking results more accurate. The CDOCKER method is used for molecular docking, and attention is paid to the CDOCKER ENERGY score and hydrogen bonds of the docking result. The CDOCKER ENERGY score represents the binding ability of ligand and receptor. A positive score indicates that ligand and receptor can bind, and the higher the score, the better the binding ability. Part of the docking complex and its 2D and 3D structure diagrams were further visualized, with hydrogen bonds marked in green.

### 2.3. *In Vitro* Experiment

#### 2.3.1. Chemicals and Reagents

LSH was harvested from Lingao County, Hainan Province, China, and Gefitinib (SML1657, ≥ 98% [HPLC]) was purchased from Sigma‐Aldrich. Caspase‐3/p17/p19 polyclonal antibody (catalog number: 19677‐1‐AP), Bcl 2 polyclonal antibody (catalog number: 26593‐1‐AP), BAX polyclonal antibody (catalog number: 50599‐2‐lg), and HRP‐conjugated affinipure goat antirabbit IgG (H + L) (catalog number: SA00001‐2) were purchased from Wuhan Sanying Biotechnology Co., Ltd. (Proteintech). The PE Annexin‐V apoptosis assay kit (catalog number: 559763) was purchased from BD Biotechnology Co., Ltd. (USA), and the CCK8 kit was purchased from DOJINDO (Shanghai, China). Both the complete animal protein extraction kit (order no. C51003) and the Bradford Method Protein Quantification kit (order no. C503031‐1000) were purchased from Sangon Biotechnology Co., Ltd. (Shanghai, China). BeyoClickEdU‐594 Cell Proliferation Test kit (product number: C0078S) and Matrix‐Gel Basement Membrane Matrix (product number: C0372) were purchased from Beyotime Biotechnology Co., Ltd. (Shanghai, China). RPMI1640 medium was purchased from Gibco, and penicillin–streptomycin–amphotericin B mixed solution (catalog number: P7630) was purchased from Solarbio Science & Technology Co., Ltd. (Beijing, China).

#### 2.3.2. Cell Culture and Drug Treatment

Human NSCLC cell lines (A549 cells, H1299 cells, PC‐9 cells) and normal human embryonic lung cells (MRC‐5 cells) were purchased from the Cell Bank of the Chinese Academy of Sciences (Shanghai, China). A549 cells, H1299 cells, and PC‐9 cells were cultured in RPMI 1640 medium containing 10% fetal bovine serum (FBS) and 1% penicillin–streptomycin–amphotericin B mixed solution, while MRC‐5 cells were cultured in MEM medium containing 10% FBS and 1% penicillin–streptomycin–amphotericin B mixed solution. All cells were cultured in an incubator at 5% CO_2_ and a temperature of 37°C.

#### 2.3.3. CCK‐8 Assay

Cell viability, which refers to the proliferation of cells, was measured using cell counting Kit 8 (CCK‐8). A549 cells (2.5 × 10^3^/well), H1299 cells (2.5 × 10^3^/well), PC‐9 cells (3.5 × 10^3^/well), and MRC‐5 cells (5 × 10^3^/well) were placed in 96‐well plates, respectively. After 24 h of culture, the cell volume was about 5 × 10^3^/well. Each well was filled with different concentrations of LSH (25, 50, 100, 200, 400, 800, 1600, and 3200 μg/mL) or gefitinib (0.375, 0.75, 1.5, 3, and 6 μM) and was applied to treat the 96‐well plates of H1299 cells, PC‐9 cells, and MRC‐5 cells, which were continued for 48 h, and the 96‐well plates of A549 cells were continued for different times (24 h, 48 h, and 72 h). 10 μL CCK‐8 solution was added to each well and incubated at 37°C for 1–4 h until the color of the untreated control cells turned orange. The absorbance (A) of the 96‐well plate at 450 nm was detected, and the IC_50_ value was calculated. Each experiment was carried out independently at least three times, and the experimental procedures were carried out according to the CCK‐8 reagent specification and references [17].

#### 2.3.4. Clone Formation Experiment

A549 cells were inoculated into 6‐well plates at a density of 600 cells/well [12], cultured for 48 h, treated with different concentrations of LSH (300, 700, and 900 μg/mL) for 48 h, and then the cells were cultured with drug‐free fresh medium for 7–10 days, changing the medium every 3 days. The 6‐well plate was removed, 4% paraformaldehyde was added, fixed for about 30 min, 0.1% crystal violet staining solution was added, cells were stained for 20 min, cleaned with ddH_2_O, dried at room temperature, photographed and stored, and the number of cell clones was counted. All experiments were performed in triplicate.

#### 2.3.5. Cell Scratch Healing Test

The cell migration ability was assessed using the wound healing assay. A549 cells (50 × 10^4^/well) were inoculated into a 6‐well plate. After 24 h of culture, the cells grew to 90%–100%. A straight line was drawn into the hole of the 6‐well plate with the tip of a 20 μL pipette gun as a wound. The original medium was discarded, and the medium containing 2% FBS was added and treated with different concentrations of LSH (700 and 900 μg/mL), respectively. The migration of cells was observed under a microscope, and images were collected at different time points (0, 24, and 48 h). The experimental procedure was referred to literature, and the migration area of cells was calculated using ImageJ_v1.8.0 software. Consider “all experiments were performed in triplicate.”

#### 2.3.6. Transwell Test

The invasion ability of A549 cells was detected by the Transwell invasion assay. The Transwell chamber was used in the experiment; the chamber was placed on a 24‐well plate, and Matrix‐GEL Matrix glue was evenly spread on the upper chamber of the chamber. A549 cells (2.5 × 10^4^/well) were inoculated with 10% FBS in the upper compartment and 800 μL of 10% FBS in the lower compartment. Culture for 24 h, the upper cavity medium was changed to the medium without FBS, treated with different concentrations of LSH (700 and 900 μg/mL), and continued to culture for 48 h. The Transwell chamber was removed, the matrix glue and cells in the upper chamber were gently wiped with a cotton swab, 600 μL of 4% paraformaldehyde was added into the 24‐well plate, and the chamber was fixed for about 30 min. An appropriate amount of 0.1% crystal violet staining solution was added into the 24‐well plate, and the chamber was placed in the 24‐well plate for 10 min, and the chamber was removed and cleaned with PBS. The stained cells were dried at room temperature, and the images were observed under a microscope. The cells were counted in three randomly selected fields, and the number of cells was calculated using ImageJ_v1.8.0 software. All experiments were performed in triplicate.

#### 2.3.7. 5‐Ethynyl‐2‐Deoxyuridine (EdU) Detection

Cell proliferation was detected by the Cell Proliferation EdU Image Kit. A549 cells (5 × 10^4^/well) were inoculated into 6‐well plates, cultured for 24 h, treated with LSH of different concentrations (700 μg/mL and 900 μg/mL), and cultured for 48 h. Follow the BeyoClick EDU‐594 cell proliferation detection kit instructions for the experiment. The brief outline is to prepare 2 × EdU working liquid (20 μM), preheat 37°C 2 × EdU working liquid (20 μM) to an equal volume into the 6‐well plate, and incubate the cells in the incubator for 3 h. Add 4% of paraformaldehyde (P0099) and fix it at room temperature for 15 min, add 1 mL of permeable solution per well (P0097 for immunostaining power), incubate at room temperature for 15 min, prepare click reaction solution according to the instructions, add 0.5 mL of click reaction solution per well, and incubate at room temperature for 30 min away from light. Add 1 mL of 1 × Hoechst 33342 solution to each well and incubate at room temperature for 10 min away from light. The images were observed and collected under a fluorescence microscope (OLYMPUS, Japan), and the percentage of EdU positive cells in each treatment group was calculated using ImageJ_v1.8.0 software. All experiments were performed in triplicate.

#### 2.3.8. Annexin‐V/7‐AAD Double Staining Flow Cytometry

A549 cells (3.0 × 10^5^/bottle) and MRC‐5 cells (5.0 × 10^5^/bottle) were inoculated in 25 cm culture bottles for 24 h and treated with different concentrations of LSH (700 μg/mL, 900 μg/mL) or gefitinib (10 μM) for 48 h, respectively. The cells were collected and transferred to a 2 mL Eppendorf microcentrifuge tube and washed with precooled PBS twice. The procedure was performed according to the instructions of the PE Annexin‐V apoptosis detection kit. Annexin‐V and 7AAD were added, and the Annexin‐V and 7AAD were cultured at room temperature without light for 15 min, then analyzed by flow cytometry (ACEANovocyte). The apoptosis rate of cells in each group = (early apoptotic cells + late apoptotic cells)/normal cells × 100% [15].

#### 2.3.9. Western Blotting Analysis

Mouse tumor tissues or A549 cells (3 × 10^5^/bottle) were inoculated in a 25 cm culture bottle for 24 h and treated with LSH (700 and 900 μg/mL) or gefitinib (10 μM) at different concentrations for 48 h. The protein was extracted according to the instructions of the animal total protein extraction kit (order no. C51003). The protein concentration of each group was detected with the Bradford method protein quantitative detection kit. SDS–PAGE loading buffer was added to the protein and denatured at 100°C for 8 min. The protein was isolated by electrophoresis with 4%–20% high resolution prefabricated adhesive (Hepes‐Tris). After electrophoresis, the protein was transferred to a PVDF membrane. It was closed with a protein‐free rapid sealing solution at room temperature for 30 min and then incubated with primary antibody at 4°C overnight and then incubated with secondary antibody at 37°C for 1 h. Finally, ECL supersensitive luminescent solution was used to display the bands on the membrane, and ImageJ_v1.8.0 was used to calculate the gray values of each group of proteins.

#### 2.3.10. Microscope Observation

A549 cells (2 × 10^4^/well) were inoculated into a 6‐well plate, cultured for 24 h, treated with LSH (900 μg/mL), continued to be cultured, and the morphological changes of the cells were observed under the microscope, and the images were collected at different time points (0, 24, and 48 h).

#### 2.3.11. Electron Microscopy Observation

A549 cells (3 × 10^5^/bottle) were inoculated in a 25 cm culture vial for 24 h and treated with LSH (900 μg/mL) or gefitinib (10 μM) for 48 h, respectively. Trypsin was added, and the cells were digested, inhaled into a centrifuge tube, and centrifuged at a low speed (2000 rpm, 10 min). The supernatant was discarded. Glutamic acid fixative that was restored to room temperature in advance was added. The cell masses were blown and suspended, fixed at room temperature for 30 min away from light, and then transferred to 4°C for overnight observation by electron microscope. Experimental methods References.

### 2.4. *In Vivo* Experiment

#### 2.4.1. Xenotransplantation and Treatment of Mouse Tumors

BALB/c‐nu male mice (3 weeks old, 14 ± 2 g) were purchased from Hunan Lake Jingda Laboratory Animal Co., Ltd. (Hunan, China). The mice were kept in iron cages under standard conditions (temperature 24 ± 1°C, humidity 50 ± 5%, light/dark cycle 12/12 h). A549 cell suspension (2 × 10^6^; 100 μL; matrix colloid volume: cell volume = 1:1) was injected into the subcutaneous xenotransplantation model of A549 cells in nude mice. The 20 mice were randomly divided into 4 groups with 5 mice in each group. After the model was established, the transplanted tumor size was measured with vernier calipers every 2–3 days and recorded. After 11 days of inoculation, the tumor diameter was ≥ 0.5 cm, indicating that the model was successfully established. The daily intragastric dosage was as follows: control group (Group A, PBS, 30 μL/kg/d), LSH low concentration group (Group B, LSH‐L, 300 mg/kg/d), LSH medium concentration group (Group C, LSH‐M, 900 mg/kg/d), LSH high concentration group (Group D, LSH‐H, 2700 mg/kg/d). In the experiment, the daily dose was referenced [18], and the weight of the mice was measured every 4 days. The drug was given by intragastric administration at 6–7 p.m. every day for 14 days, and the time of administration was referenced [19]. On the 15th day, the nude mice were killed, the mice were photographed, the tumors were removed, and the tumor size and weight were measured. Calculate the tumor volume: V (mm^3^) = L (length)×W^2^ (width)/2. This study has been reviewed and approved by the Experimental Ethics Committee of Hainan Medical University (Ethics review number: HY2022‐026), and the animal experiment meets the welfare and ethical requirements of experimental animals, as well as the requirements of the animal breeding environment for scientific research. Part of the tumor tissues of each group of mice were cleaned and stored in the −80°C refrigerator for lysis, and Western blot analysis was performed. The other part of the tumor tissue was fixed in 4% paraformaldehyde, embedded in paraffin, sectored, and stained with HE and immunohistochemical (IHC) staining [20].

#### 2.4.2. Detection of Pathological Changes of Mouse Organs and Serum Biomarkers

Different organs (lung, liver, heart, and kidney) of each group of mice were collected and fixed in 4% paraformaldehyde, embedded in paraffin, sliced, and stained with HE, and the pathological changes of each organ tissue were observed under the microscope. A serum separation tube was used to collect the blood of each group of mice. The serum separation tube was placed at 4°C overnight, centrifuged at low speed (1000 rpm, 20 min), and the serum was taken as serum. The serum was sent to Wuhan Xville Biotechnology Co., Ltd. for detection of liver function biomarkers, alanine aminotransferase (ALT), aspartate aminotransferase (AST), and renal function biomarkers, creatinine (CREA), and blood urea nitrogen (BUN) [21].

### 2.5. Statistical Analysis

Using GraphPad Prism Version 10.1.2 statistical analysis software for data and map statistics, experimental data are expressed as a mean ± standard deviation (SD). When there were only 2 groups of data, *T*‐test analysis was used for intergroup comparison; when there were multiple groups of data, one‐way analysis of variance was used for intergroup comparison, and *p* < 0.05 was considered statistically significant.

## 3. Results

### 3.1. Chemical Composition Analysis of LSH

UHPLC–OE–MS was used for the chemical composition analysis of LSH. After the original data were converted into mzXML format by ProteoWizard software, metabolites were identified using the Collaborative R package. The databases used were BiotreeTCM (V1.0) and BT‐HERB (V1.0). Then use the independently written R package for visual analysis. More than 1000 compounds were identified from the medicinal liquid samples extracted from the LSH water extracted medicinal liquid sample, the specific information of which included MS2_name, Formula, pubchemid, MS2_score, and others, and the full information of all substances detected by LSH (see supporting data excel table: data). According to literature reports, 19 ingredients with Magnoflorine and OB values ≥ 30%, DL values ≥ 0.18, and MS2 score values were selected, and a total of 20 active ingredients were screened out (Table 1). The TIC map was detected by positive ion mode, and 9 active ingredients were shown as peak samples (Figure 1A). The TIC diagram was detected by negative ion mode, and 11 active components were labeled with peaks (Figure 1B).

**TABLE 1:** 20 pharmaceutical active ingredients of LSH.

TIC No.	MS2_name	Formula	Pubchem ID	Structure	OB (%)	DL	Subclass	MS2_score	Rtmed
1	*trans*‐11‐Eicosenoic acid	C20H38O2	5282769	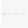	30.7	0.2	Unsaturated fatty acids	3.96	590.3
2	Skimmin	C15H16O8	99693	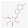	38.35	0.32	Simple coumarins	3.96	234.7
3	*cis*‐8,11,14‐Eicosatrienoic acid	C20H34O2	5280581	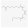	44.11	0.2	Unsaturated fatty acids	3.95	558.3
4	*cis*‐11‐Eicosenoic acid	C20H38O2	5282768	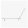	30.7	0.2	Unsaturated fatty acids	3.94	590.3
5	N‐trans‐Feruloyltyramine	C18H19NO4	5280537	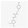	86.71	0.26	Cinnamic acid amides	3.92	325.2
6	Aurantiamide acetate	C27H28N2O4	124319	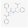	58.38	0.52	Dipeptides	3.91	421.9
7	Baicalin	C21H18O11	64982	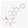	40.12	0.75	Flavones	3.9	326.9
8	Boldine	C19H21NO4	10154	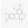	31.18	0.51	Aporphine alkaloids	3.89	236.3
9	Kaempferol 3‐methyl ether	C16H12O6	5280862	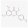	60.16	0.26	Flavonols	3.87	376.5
10	(S)‐Scoulerine	C19H21NO4	439654	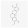	32.28	0.54	Isoquinoline alkaloids	3.86	236.3
11	Aurantiamide	C25H26N2O3	185904	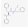	45.76	0.43	Dipeptides	3.86	402.3
12	Machiline	C17H19NO3	440989	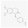	79.64	0.24	Isoquinoline alkaloids	3.86	231.3
13	Glycitein	C16H12O5	5317750	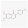	50.48	0.24	Isoflavones	3.82	365.9
14	Pratensein	C16H12O6	5281803	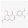	39.06	0.28	Isoflavones	3.81	365.1
15	7‐Methoxybaicalein	C16H12O5	471719	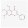	41.16	0.23	Flavones	3.8	365.9
16	Calycosin	C16H12O5	5280448	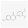	47.75	0.24	Isoflavones	3.77	365.9
17	Genkwanin	C16H12O5	5281617	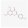	37.13	0.24	Flavones	3.75	365.9
18	Acacetin	C16H12O5	5280442	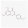	34.97	0.24	Flavones	3.74	365.9
19	Eriodictyol	C15H12O6	440735	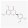	71.79	0.24	Flavanones	3.7	371.9
20	Magnoflorine	C20H24NO4	73337	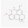	26.69	0.55	Aporphine alkaloids	2.73	291.3

**FIGURE 1:**
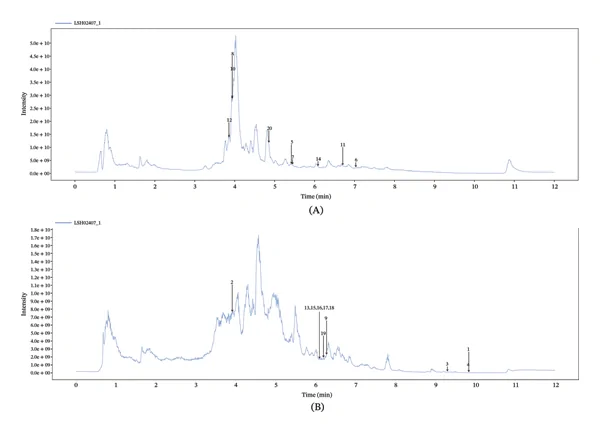
UHPLC–OE–MS testing result of Long Stephania Herb (LSH). (A) Positive ion mode TIC detection diagram, 9 active ingredients (Peak 5: N‐trans‐feruloyltyramine; Peak 6: aurantiamide acetate; Peak 7: baicalin; Peak 8: boldine; Peak 10: (S)‐scoulerine; Peak 11: aurantiamide; Peak 12: glycitein; Peak 14: pratensein; Peak 20: magnoflorine). (B) TIC diagram detected in negative ion mode with 11 active ingredients (Peak 1: trans‐11‐eicosenoic acid; Peak 2: skimmin; Peak 3: cis‐8,11,14‐eicosatrienoic acid; Peak 4: cis‐11‐eicosenoic acid; Peak 9: kaempferol 3‐methyl ether; Peak 13: glycitein; Peak 15: 7‐methoxybaicalein; Peak 16: calycosin; Peak 17: genkwanin; Peak 18: acacetin; Peak 19: eriodictyol).

### 3.2. LSH Morphology and Potential Molecular Targets for Treatment of NSCLC

LSH grows in Hainan, southeastern Yunnan, Guangxi, Guangdong, Fujian, and Taiwan in China. LSH mostly grows in dry places in mountainous areas and sparse forests and is often intertwined with shrubs. Its leaf surface shape is triangular oval, with blunt leaf tips, and the shape is just like the dustpan used to hold manure, so it is named (Figure 2A). There were a total of 576 therapeutic targets with probability > 0 obtained from the SwissTargetPrediction database for 20 active ingredients. 2034 related targets in NSCLC with scores > 25 were obtained from the GeneCards database, 479 related targets in NSCLC were obtained from the OMIM database, and 2434 targets with high correlation with NSCLC were obtained by removing overlapping targets from two databases. A total of 261 crossover targets between LSH and NSCLC were screened by Venn diagram (Figure 2B), which were potential targets specifically related to LSH treatment of NSCLC. The PPI network was constructed by inputting cross‐targets into the STING12.0 database. 256 nodes and 2108 edges were obtained, and the average node‐degree value was 16.2. Construct a visually optimized PPI network diagram using the network analysis tool in Cytoscape software (Figure 2C). The size and color brightness of nodes correspond to their respective degree values. The larger the node and the brighter the color, the higher the node degree value. The dimension thickness and color brightness of an edge are proportional to its binding score, which indicates that the larger the size of an edge and the brighter the color, the higher the binding score. The CentiScape2.2 plug‐in in Cytoscape software was used to screen core targets according to betweenness, closeness, and degree, and 48 core targets for the treatment of NSCLC by LSH were determined (Table 2). These core targets play a key role in LSH’s anti‐NSCLC effect. The core target network was analyzed using the networkAnalysis tool, and an optimized core target PPI network was created, which has 48 nodes and 543 edges. The interaction and functional correlation between these core targets are visualized. Among these core targets, the top 10 core targets are AKT1, STAT3, EGFR, HSP90AA1, SRC, GAPDH, JUN, BCL2, CASP3, and CCND1 (Figure 2D). In the figure, the node size of these targets is larger, and the node color is redder. These targets are the key targets for LSH treatment of NSCLC. The proteins encoded by these key target genes play an important role in a variety of cellular functions, including cancer initiation and development, cell signaling, cell cycle regulation, regulation of cell proliferation, cell migration, and induction of apoptosis.

**FIGURE 2:**
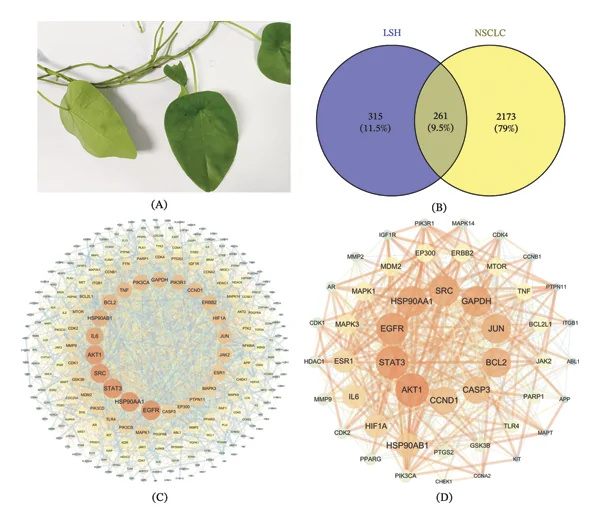
The picture of LSH, the potential target of LSH, preventing lung cancer. (A) The picture of LSH. (B) A total of 261 crossover targets between LSH and NSCLC were screened by Venn diagram, and these 261 crossover targets were potential targets specifically related to LSH treatment of NSCLC. (C) PPI network of the LSH and lung cancer overlapping targets. The size and color brightness of the nodes correspond to their respective degree values. The larger the node and the brighter the color, the higher the node degree value. The thickness and color brightness of the edges are proportional to their binding fraction. This suggests that thicker edges and brighter colors mean higher binding scores. (D) The central 48 targets the PPI network of LSH and lung cancer overlapping targets. The top 10 core targets are AKT1, STAT3, EGFR, HSP90AA1, SRC, GAPDH, JUN, BCL2, CASP3, and CCND1.

**TABLE 2:** 48 core targets screened from the PPI network for the treatment of NSCLC by LSH.

No.	Gene name	Degree	Betweenness	Closeness	Topological coefficient
1	AKT1	76	3680.598756	0.002173913	0.50245499
2	STAT3	80	4113.562886	0.002169197	0.51399776
3	EGFR	80	4018.718313	0.002192982	0.50258769
4	GAPDH	55	4408.86257	0.00209205	0.52423168
5	SRC	77	3837.608552	0.002150538	0.50827423
6	HSP90AA1	80	4153.474794	0.002188184	0.51477541
7	BCL2	59	2447.984915	0.002070393	0.5331307
8	JUN	51	1000.134463	0.00203666	0.5331307
9	CASP3	43	788.4238116	0.001953125	0.50290135
10	CCND1	54	1847.556136	0.00203252	0.53320438
11	HSP90AB1	62	1948.110362	0.002061856	0.54083734
12	HIF1A	51	1993.743914	0.002024291	0.56170213
13	ESR1	47	2121.897903	0.001996008	0.55392517
14	IL6	68	5474.481669	0.002096436	0.55759354
15	MAPK3	44	1338.019152	0.001937984	0.56301146
16	MDM2	38	1048.727058	0.001851852	0.55489362
17	MAPK1	43	1094.041485	0.001930502	0.54893617
18	MTOR	35	557.3406078	0.001919386	0.59131206
19	EP300	43	1510.273159	0.001879699	0.53368794
20	ERBB2	53	2486.0853	0.001992032	0.54891304
21	TNF	57	3487.044069	0.002	0.55134135
22	BCL2L1	35	630.5783746	0.001893939	0.57350097
23	JAK2	50	1840.918399	0.001908397	0.56129686
24	TLR4	41	2125.764856	0.001897533	0.59680851
25	PARP1	33	1223.770495	0.001841621	0.54680851
26	PTGS2	31	2279.065147	0.001858736	0.59798432
27	GSK3B	38	1360.241484	0.001821494	0.58790594
28	PIK3CA	56	951.3258375	0.001915709	0.51847704
29	CDK2	35	481.6111067	0.001715266	0.54589372
30	HDAC1	29	550.3614659	0.001763668	0.60047281
31	PPARG	26	863.0575799	0.001858736	0.60401891
32	MMP9	37	1179.422903	0.001890359	0.62884161
33	CDK1	37	1381.174168	0.001733102	0.52941176
34	AR	24	651.6383205	0.001782531	0.63329161
35	IGF1R	31	1708.922156	0.001779359	0.5518617
36	MMP2	25	464.2016486	0.001845018	0.63031915
37	MAPK14	29	530.7171378	0.001811594	0.625
38	PIK3R1	54	1033.123714	0.001872659	0.5212766
39	CDK4	32	567.4177436	0.001748252	0.53989362
40	PTPN11	44	615.4354532	0.001818182	0.57142857
41	ITGB1	33	1068.857942	0.001801802	0.54968944
42	CCNB1	33	1141.710009	0.001724138	0.53495441
43	MAPT	22	657.6675186	0.001754386	0.60883797
44	APP	27	1216.150483	0.001785714	0.58756137
45	ABL1	25	1018.006574	0.00170068	0.55482815
46	KIT	24	1126.495428	0.001769912	0.58510638
47	CCNA2	30	466.0273761	0.001607717	0.46304348
48	CHEK1	27	460.2829809	0.001589825	0.50123457

### 3.3. GO Analysis and KEGG Pathway Enrichment Analysis of Cross Targets and Observation of Treatment for NSCLC

GO analysis of 256 targets in the PPI network using the Metscape database (*p* < 0.01) showed that 2497 BP, based on the lowest *p* value. The top 5 BPs are phosphorylation, protein phosphorylation, positive regulation of phosphorus metabolic process and positive regulation of phosphate metabolic process, and positive regulation of phosphorylation; 157 cell components (CCs), based on the lowest *p* value, The top 5 CCs are receptor complex, protein kinase complex, and transferase complex. Transferring phosphorus‐containing groups, serine/threonine protein kinase complex, and side of membrane; 264 MFs, based on the lowest *p* value; the top 5 MFs bits are protein kinase activity, phospho transferase activity, alcohol group as acceptor, kinase activity, protein tyrosine kinase activity, and protein serine/threonine kinase activity. The top 15 items with the smallest *p*‐value in BP, CC, and MF were selected to make the classification histogram. The *p*‐value reflected the statistical significance of enrichment, and the smaller the *p*‐value, the higher significance. The height of each bar corresponds to the number of enriched genes, and the greater the bar height, the more genes were enriched (Figure 3A). These enriched LSH highlight key BP, CCs, and MFs in NSCLC. After KEGG pathway analysis, 209 signaling pathways were obtained, with statistical significance (*p* < 0.01). 20 KEGG pathways with a low *p*‐value and associated with NSCLC were selected to generate a statistical bubble map of significance (Figure 3B). Each bubble represents a specific pathway, and the *p*‐value indicates the importance of the enrichment pathway. The more important pathway is, and count represents the number of genes enriched. The larger value of count is, the more genes are enriched, and the larger the bubble area. The specific information of the 20 signal paths was compiled into a table (Table 3). The top two signal paths are pathways in cancer and the PI3K–Akt signaling pathway suggest that LSH is potentially associated with various cancers, and LSH is highly likely to play an anti‐NSCLC role through the PI3K–Akt signaling pathway. In addition, the MAPK signaling pathway, the Ras signaling pathway, and the JAK–STAT signaling pathway are all closely related to LSH anti‐NSCLC.

**FIGURE 3:**
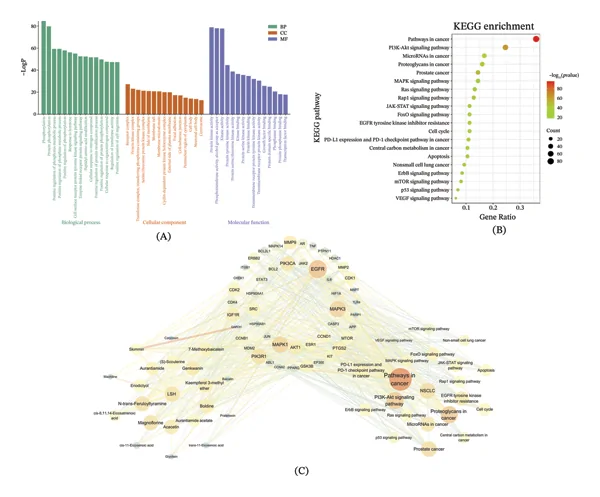
GO and KEGG enrichment analysis, LSH‐active component–core target–pathway–NSCLC network diagram. (A) Cross‐target GO analysis (biological processes, cell components, and molecular functions). Histogram of the top 15 enrichment items (*p* < 0.01), the *p* value shows the statistical significance of enrichment, and the smaller the *p*‐value, the higher the significance. The height of each bar corresponds to the number of enriched genes, and the greater the bar height, the more enriched genes. (B) Cross‐target KEGG pathway analysis; important 20 pathways bubble map (*p* < 0.01); the *p*‐value indicates the importance of the enrichment pathway; the smaller the *p* value, the redder the bubble color, and the more important the pathway. Count represents the number of genes enriched; the larger the count value, the more genes are enriched, and the larger the bubble area. (C) LSH‐active component–core target–pathway–NSCLC network diagram. The network diagram has 90 nodes and 534 edges. The size and color brightness of each point in the diagram are determined by the degree value. The larger the degree value, the larger the point, and the brighter the color.

**TABLE 3:** The information of 20 KEGG pathways and their correspondent core target genes is regulated by pharmaceutical active ingredients of LSH.

No.	KEGG ID	KEGG pathway	Core target gene	Gene count	*p* value
1	hsa05200	Pathways in cancer	ABL1; AKT1; AR; CCND1; BCL2; BCL2L1; CASP3; CCNA2; CDK2; CDK4; EGFR; EP300; ERBB2; ESR1; MTOR; GSK3B; HDAC1; HIF1A; HSP90AA1; HSP90AB1; IGF1R; IL6; ITGB1; JAK2; JUN; KIT; MDM2; MMP2; MMP9; PIK3CA; PIK3R1; PPARG; MAPK1; MAPK3; PTGS2; STAT3	92	1.72606E − 95
2	hsa04151	PI3K–Akt signaling pathway	AKT1; CCND1; BCL2; BCL2L1; CDK2; CDK4; EGFR; ERBB2; MTOR; GSK3B; HSP90AA1; HSP90AB1; IGF1R; IL6; ITGB1; JAK2; KIT; MDM2; PIK3CA; PIK3R1; MAPK1; MAPK3; TLR4	63	2.24167E − 64
3	hsa05206	MicroRNAs in cancer	ABL1; CCND1; BCL2; CASP3; EGFR; EP300; ERBB2; MTOR; HDAC1; MDM2; MMP9; PIK3CA; PIK3R1; MAPK1; MAPK3; PTGS2; STAT3	43	3.61864E − 39
4	hsa05205	Proteoglycans in cancer	AKT1; CCND1; CASP3; MAPK14; EGFR; ERBB2; ESR1; MTOR; HIF1A; IGF1R; ITGB1; MDM2; MMP2; MMP9; PIK3CA; PIK3R1; MAPK1; MAPK3; PTPN11; SRC; STAT3; TLR4; TNF	41	2.09281E − 44
5	hsa05215	Prostate cancer	AKT1; AR; CCND1; BCL2; CDK2; EGFR; EP300; ERBB2; MTOR; GSK3B; HSP90AA1; HSP90AB1; IGF1R; MDM2; MMP9; PIK3CA; PIK3R1; MAPK1; MAPK3	37	1.08787E − 51
6	hsa04010	MAPK signaling pathway	AKT1; CASP3; MAPK14; EGFR; ERBB2; IGF1R; JUN; KIT; MAPT; MAPK1; MAPK3; TNF	37	6.21012E − 32
7	hsa04014	Ras signaling pathway	ABL1; AKT1; BCL2L1; EGFR; IGF1R; KIT; PIK3CA; PIK3R1; MAPK1; MAPK3; PTPN11	34	1.3444E − 31
8	hsa04015	Rap1 signaling pathway	AKT1; MAPK14; EGFR; IGF1R; ITGB1; KIT; PIK3CA; PIK3R1; MAPK1; MAPK3; SRC	33	5.86743E − 32
9	hsa04630	JAK–STAT signaling pathway	AKT1; CCND1; BCL2; BCL2L1; EGFR; EP300; MTOR; IL6; JAK2; PIK3CA; PIK3R1; PTPN11; STAT3	29	1.53205E − 29
10	hsa04068	FoxO signaling pathway	CCND1; CCNB1; CDK2; MAPK14; EGFR; EP300; IGF1R; IL6; MDM2; PIK3CA; PIK3R1; MAPK1; MAPK3; STAT3	29	1.08685E − 32
11	hsa01521	EGFR tyrosine kinase inhibitor resistance	AKT1; BCL2; BCL2L1; EGFR; ERBB2; MTOR; GSK3B; IGF1R; IL6; JAK2; PIK3CA; PIK3R1; MAPK1; MAPK3; SRC; STAT3	29	5.10765E − 40
12	hsa04110	Cell cycle	ABL1; CCND1; CCNA2; CCNB1; CDK1; CDK2; CDK4; CHEK1; EP300; GSK3B; HDAC1; MDM2	29	2.33698E − 30
13	hsa05235	PD‐L1 expression and PD‐1 checkpoint pathway in cancer	AKT1; MAPK14; EGFR; MTOR; HIF1A; JAK2; JUN; PIK3CA; PIK3R1; MAPK1; MAPK3; PTPN11; STAT3; TLR4	28	1.85709E − 36
14	hsa05230	Central carbon metabolism in cancer	EGFR; ERBB2; MTOR; HIF1A; KIT; PIK3CA; PIK3R1; MAPK1; MAPK3	27	5.31364E − 38
15	hsa04210	Apoptosis	PARP1; AKT1; BCL2; BCL2L1; CASP3; JUN; PIK3CA; PIK3R1; MAPK1; MAPK3; TNF	27	2.6146E − 29
16	hsa05223	Nonsmall cell lung cancer	AKT1; CCND1; CDK4; EGFR; ERBB2; PIK3CA; PIK3R1; MAPK1; MAPK3; STAT3	24	4.65182E − 32
17	hsa04012	ErbB signaling pathway	ABL1; AKT1; EGFR; ERBB2; MTOR; GSK3B; JUN; PIK3CA; PIK3R1; MAPK1; MAPK3; SRC	22	1.00433E − 26
18	hsa04150	mTOR signaling pathway	AKT1; MTOR; GSK3B; IGF1R; PIK3CA; PIK3R1; MAPK1; MAPK3; TNF	21	3.20335E − 19
19	hsa04115	p53 signaling pathway	CCND1; BCL2; BCL2L1; CASP3; CCNB1; CDK1; CDK2; CDK4; CHEK1; MDM2	18	1.60328E − 21
20	hsa04370	VEGF signaling pathway	MAPK14; PIK3CA; PIK3R1; MAPK1; MAPK3; PTGS2; SRC	16	4.42828E − 20

The LSH‐active components–core target–pathway–NSCLC visual network diagram (Figure 3C) is constructed using Cytoscape software. The network diagram has 90 nodes and 534 edges. The size and color brightness of each dot in the diagram are determined by the degree value. The NetworkAnalysis tool was used to analyze the network and found that the average node degree was 11.80. The network topology analysis results show that N‐trans‐feruloyltyramine, magnoflorine, genkwanin, acacetin, boldine, kaempferol 3‐methylether, aurantiamide, 7‐methoxybaicalein (S)‐scoulerine, eriodictyol, and aurantiamideacetate were 11 key effective compounds in LSH, and the active compounds N‐trans‐feruloyltyramine and magnoflorine had the highest node‐degree values. 18 core target nodes were connected, respectively. In the results of network topology analysis, the top 10 core targets include EGFR, MAPK3, MAPK1, PIK3R1, PIK3CA, AKT1, and SRC. The network topology analysis results showed that the LSH anti‐NSCLC cellular signaling pathways mainly include the PI3K/Akt signaling pathway, proteoglycans in cancer, EGFR tyrosine kinase, and the MAPK signaling pathway. This suggests that LSH may be a multicomponent, multitarget, and multipathway for anti‐NSCLC.

### 3.4. Molecular Docking Results and Analysis

Molecular docking analysis is carried out by DiscoveryStudio2022 software. The interactions between the 11 key active compounds of LSH and their predicted corresponding 10 key core targets (EGFR, PIK3CA, PIK3R1, AKT1, HSP90AA1, SRC, MAPK1, MAPK3, JUN, and CASP3) were investigated. The results showed that each key effective compound formed a relatively stable complex with its key core target corresponding to its prediction. N‐trans‐Feruloyltyramine and EGFR (Figure 4A), aurantiamide acetate and PIK3CA, the result showed that aurantiamide acetate can bind to amino acid residues on the PIK3CA molecule, such as Ser774 and Asp933 (Figure 4B), eriodictyol and AKT1 (Figure 4C), (S)‐scoulerin and HSP90AA1 (Figure 4D), eriodictyol and SRC (Figure 4E), and magnoflorine and CASP3 (Figure 4F). The scores of CDOCKER ENERGY were all positive (Table 4), indicating that these 10 proteins may be potential binding targets of key effective compounds of LSH, and the molecular docking level verified that LSH can target ant‐NSCLC by a multitarget approach.

**FIGURE 4:**
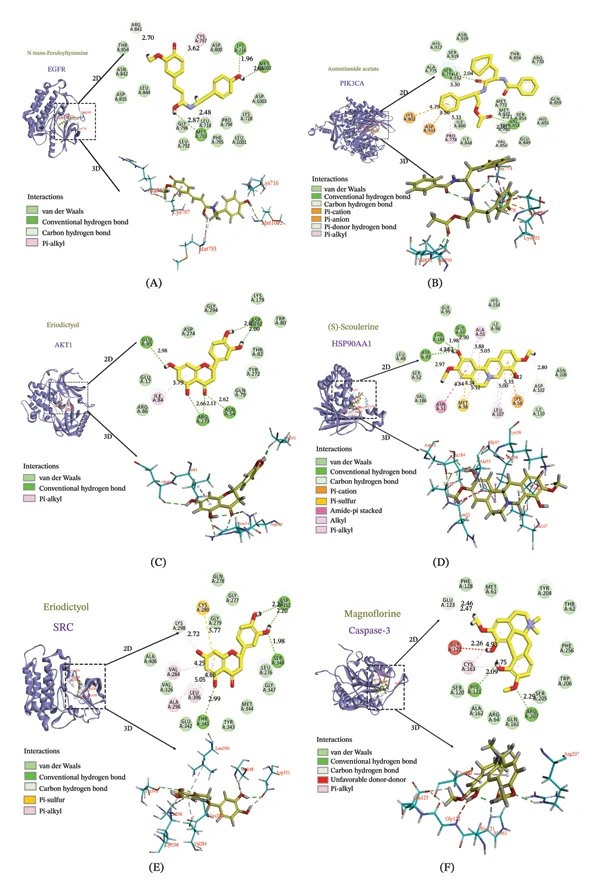
The representative compounds of LSH molecular docking with each target protein. (A) N‐trans‐Feruloyltyramine and EGFR; (B) aurantiamide acetate and PIK3CA; (C) eriodictyol and AKT1; (D) (S)‐scoulerin and HSP90AA1; (E) eriodictyol and SRC; and (F) magnoflorine and CASP3.

**TABLE 4:** Molecular docking of 11 key core components with 10 predicted core targets which interact with pharmaceutical active ingredients of LSH.

SwissTargetPrediction	PDB ID	LSH compound	CDOCKER ENERGY	SBD site sphere coordinates	SBD site sphere radius
EGFR	3IKA	N‐trans‐Feruloyltyramine	32.4064	*X* = −12.4188 *Y* = 12 16.287016 *Z* = 32.142447	18.61301
		Boldine	6.31301		
		Kaempferol 3‐methyl ether	35.2476		
		7‐Methoxybaicalein	26.5791		
		Genkwanin	28.1816		
		Acacetin	26.5272		
		Magnoflorine	5.05628		

PIK3CA	6GVF	Aurantiamide acetate	49.561	*X* = −17.054102 *Y* = 147.085708 *Z* = 28.571664	18.61301

PIK3R1	6D81	Kaempferol 3‐methyl ether	8.08925	*X* = −0.784600 *Y* = 4.131600 *Z* = −6.578800	18.61301
		Acacetin	12.1863		

AKT1	6HHF	Kaempferol 3‐methyl ether	31.4507	*X* = 4.427584 *Y* = 5.719844 *Z* = 13.257373	18.61301
		Eriodictyol	34.2526		

HSP90AA1	3O0I	N‐trans‐Feruloyltyramine	34.6631	*X* = −2.421588 *Y* = −10.599783 *Z* = −23.825001	18.61301
		Boldine	15.9808		
		(S)‐Scoulerine	26.1375		

SRC	6 E6E	Boldine	16.8696	*X* = 35.269367 *Y* = 3.247008 *Z* = 10.187130	18.61301
		Kaempferol 3‐methyl ether	26.5046		
		(S)‐Scoulerine	22.376		
		Aurantiamide	40.126		
		7‐Methoxybaicalein	31.8179		
		Genkwanin	30.984		
		Acacetin	27.9586		
		Eriodictyol	34.0809		
		Magnoflorine	5.82867		

MAPK1	6G54	Boldine	18.4524	*X* = 69.204995 *Y* = 15.345665 *Z* = 10.658459	18.61301
		Aurantiamide	48.4189		
		Magnoflorine	5.37002		

MAPK3	6GES	Genkwanin	43.5794	*X* = 61.670151 *Y* = 21.486308 *Z* = 18.132958	18.61301
		Magnoflorine	7.46409		

C‐JUN	6Y3V	(S)‐Scoulerine	14.8792	*X* = 17.635708 *Y* = −14.832954 *Z* = −9.502612	18.61301

CASP3	3J32	Magnoflorine	0.532294	*X* = 37.499888 *Y* = 11.736655 *Z* = 67.752064	18.61301

### 3.5. LSH Inhibits the Growth and Invasion of NSCLC

In the CCK‐8 assay, LSH significantly reduced the viability of NSCLC cells (A549, H1299, and PC‐9) in a dose‐dependent manner. The IC50 of A549 cells, H1299 cells, and PC‐9 cells after LSH treatment for 48 h were 277.1, 504.0, and 603.1 μg/mL, respectively. The IC50 of normal embryonic lung cells (MRC‐5 cells) after treatment with LSH for 48 h was 975.3 μg/mL, which was more than 1.5–3 times that of NSCLC cells, indicating that LSH had selective cytotoxic effects on NSCLC cells (Figure 5A). In the positive control experiment, the IC50 of NSCLC cells (A549, H1299, and PC‐9 cells) and normal embryonic lung cells (MRC‐5 cells) after gefitinib treatment for 48 h were 1.757, 7.161, 1.627, and 22.51 μM, respectively (Figure 5A). In addition, LSH significantly reduced the viability of A549 cells in a dose‐ and time‐dependent manner, and the IC50 of A549 cells after treatment with LSH for 24, 48, and 72 h was 641.5, 277.1, and 182.2 μg/mL, respectively (Figure 5B). The antiproliferation effect of LSH on A549 cells was detected in the clonal formation experiment and EdU cell proliferation assay. The experimental results showed that compared with the control group (untreated), LSH significantly reduced the number of A549 cell colonies (Figure 5C) and the percentage of EdU‐positive cells in a dose‐dependent manner (Figure 5D). Results of wound healing experiments showed that LSH significantly inhibited A549 cell migration in a dose‐dependent manner compared with the control group (*p* < 0.01) (Figure 5E). Transwell cell invasion assay results also showed that LSH significantly inhibited A549 cell invasion in a dose‐dependent manner compared with the control group (*p* < 0.01) (Figure 5F).

**FIGURE 5:**
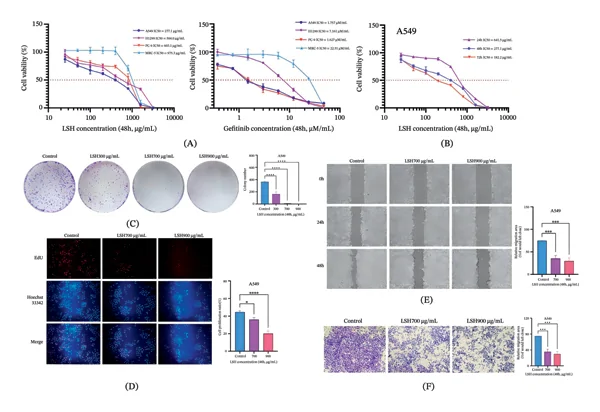
LSH inhibited the proliferation, migration, and invasion of NSCLC cells. (A) LSH inhibited the proliferation of A549 cells, H1299 cells, PC‐9 cells, and MRC‐5 cells; Gefitinib was used as a positive control (*n* = 3). (B) LSH significantly inhibited A549 cell viability in a time‐dependent manner (*n* = 3). (C) LSH reduced the number of A549 cell colonies in a dose‐dependent manner (*n* = 3). (D) LSH decreased the percentage of EdU positive cells in A549 cells in a dose‐dependent manner (*n* = 3). (E) LSH restrained A549 cell migration in a dose‐dependent manner. (F) LSH suppressed A549 cell invasion in a dose‐dependent manner (*n* = 3). Each group of data is presented as the mean ± SD, ^∗^ *p* < 0.05, ^∗∗∗^ *p* < 0.01, ^∗∗∗∗^ *p* < 0.001.

### 3.6. LSH Promotes Apoptosis of NSCLC Cells

Annexin‐V/7‐AAD double staining was used to measure the apoptosis rate of A549 cells. Compared with the control group (untreated), the Annexin‐V positive cells and 7‐AAD positive cells in the LSH‐treated groups and the gefitinib‐positive control groups significantly increased the apoptosis rate of A549 cells, indicating that LSH possesses a medicinal activity to promote apoptosis of NSCLC cells in a dose‐dependent manner (Figure 6A). In the MRC‐5 cell, the apoptosis rate of cells in the LSH treated group was not significantly increased compared with the control group (untreated) (Figure 6B). Microscopic observation showed that the cell shape of A549 cells in the LSH treated group was round and oval, and the cells lost the original polygonal cell shape. Some cells swollen and nuclei shrank in size and nucleus fragmentation; these were considered as apoptosis, while some cells swollen and nuclei concentrated were considered as pyroptosis (Figure 6C). Transmission electron microscopy (TEM) observation showed that the cells in the control group (untreated) were less damaged and a few organelles vacuolated. The cells in the LSH‐treated group emerged, and the membrane bubbled out. The results of these electron microscopy observations suggest that these cells may be in the early stage of pyroptosis. The cells of the gefitinib‐treated group showed cellular matrix concentration, although the key biomarkers of ferroptosis have not yet been detected in this experiment; electron microscopy results revealed mitochondrial condensation, a hallmark feature of ferroptosis. Therefore, we hypothesize that treatment with gefitinib may induce ferroptosis in NSCLC cells (Figure 6D). Western blotting results showed that compared with the untreated group, the Bax/Bcl‐2 ratio in the LSH‐treated group and the gefitinib‐treated group was significantly increased. The result also indicated that LSH (900 µg/mL) was able to inhibit the expression of phosphorylated AKT (p‐AKT) of A549 cells (Figure 6E). Due to the protein levels of cleaved caspase‐3 and cleaved PARP1 being positively correlated with apoptosis, which was consistent with the effect of inducing apoptosis, and *p*‐AKT being a key molecule for activating the PI3K/AKT signaling pathway, suggesting that LSH can inhibit PI3K/AKT signaling and thereby block the proliferation of lung cancer cells. These results indicated that LSH was able to promote apoptosis of A549 cells.

**FIGURE 6:**
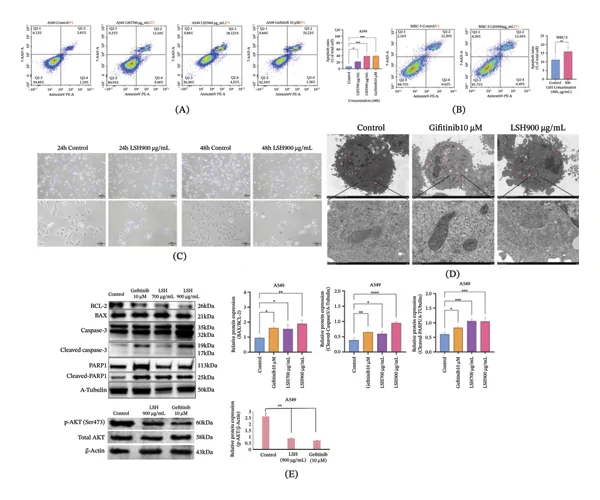
LSH stimulates the apoptosis of NSCLC cells. (A) Annexin‐V/7‐AAD double staining showed that LSH increased the apoptosis ratio of A549 cells in a dose‐dependent manner; the bar chart on the right shows the statistics of the proportion of cell apoptosis (*n* = 3). (B) Annexin‐V/7‐AAD double staining showed that LSH had no significant effect on the apoptosis of MRC‐5 cells; the bar chart on the right shows the statistics of the proportion of cell apoptosis (*n* = 3). (C) Microscopic images showed that some cells and nuclei were reduced in size and nucleus fragmentation (red arrow), which was considered apoptosis. Swelling of some cells and concentration of nuclei (black arrow) are considered as scorched cells. (D) Transmission electron microscopy (TEM) images showed that the cell was in the early stage of ferroptosis, with cell membrane vesicles (black arrow), cell membrane protrudes (PS), nuclei (N), mitochondria (M), rough endoplasmic reticulum (RER), lipid droplets (LD), lysosomes (Ly), autophagy lysosomes (ASS), cell membrane vesicles (black arrow), and a severe nuclear membrane indentation (red arrow). (E) Western blotting results showed that LSH increased the expression of cleaved caspase‐3 and cleaved PARP1 and inhibited the expression of Bcl‐2 and the ratio of Bax/Bcl‐2 protein content; and LSH (900 µg/mL) inhibited the expression of p‐AKT (Ser473) in A549 cells; the bar chart on the right shows the statistics of gray value scanning of protein bands (*n* = 3). Each group of data is presented as the mean ± SD. ^∗^ *p* < 0.05, ^∗∗^ *p* < 0.01, ^∗∗∗^ *p* < 0.001, ^∗∗∗∗^ *p* < 0.0001. The picture represents the results of three repeated experiments.

### 3.7. LSH Inhibits the Growth of Tumor in Xenografted NSCLC Animal Model

A549 cells were subcutaneously transplanted into mice to evaluate the anti‐NSCLC effect of LSH in vivo. Mice were treated with LSH by intragastric administration, including a control group (PBS, 30 μl/kg/day), an LSH low concentration group (LSH‐L, 300 mg/kg/day), an LSH medium concentration group (LSH‐M, 900 mg/kg/day), and an LSH high concentration group (LSH‐H, 2700 mg/kg/day) for 14 days (Figure 7A). The detection of tumor volume and tumor weight, the results showed that LSH significantly inhibited the growth of A549 cells in mouse xenografts (Figure 7B), and LSH inhibited the tumor volume and tumor weight in the xenograft mouse model in a dose‐dependent manner (Figure 7C). Western blotting detected the expression levels of Bax, Bcl‐2, caspase‐3, and cleaved caspase‐3 in mouse xenograft tumors. In the mouse model, the Bax/Bcl‐2 ratio was significantly higher in the LSH treatment group than in the untreated group. At the same time, caspase‐3, cleaved caspase‐3, and cleaved PARP1 protein levels increased significantly (Figure 7D). IHC staining was used to detect the expression levels of Ki‐67, caspase‐3, and Bcl‐2 in mouse xenografted tumors. The IHC analysis showed that the expressions of Ki‐67 and Bcl‐2 were significantly decreased in the LSH‐H–treated group compared with the untreated group, while the expression of caspase‐3 was increased. These results indicated that, in mouse models, LSH inhibited tumor proliferation and progression while promoting tumor cell apoptosis (Figure 7E). Implying that LSH stimulated apoptosis of NSCLC cells through activating the caspase‐3/PARP1 signaling pathway.

**FIGURE 7:**
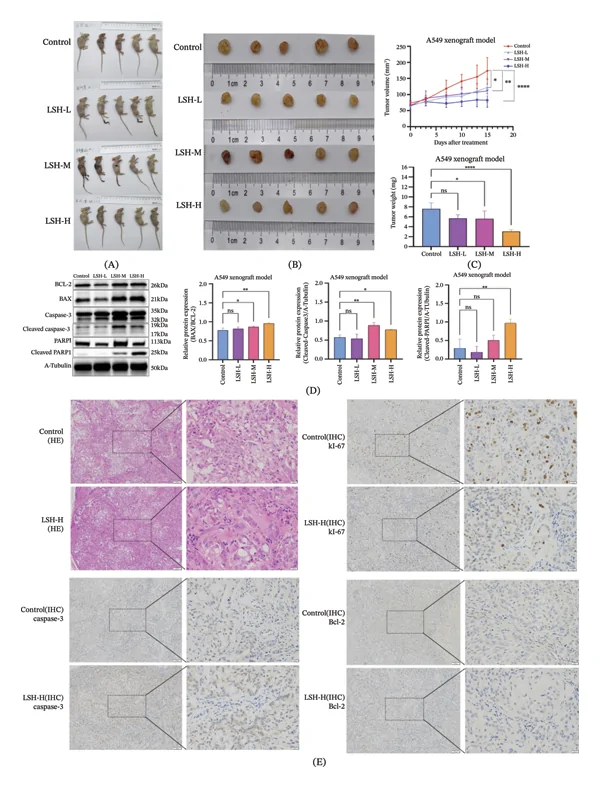
Antitumor effects of LSH on xenografted NSCLC tumors in vivo. (A) BALB/c‐nu male mice were xenografted with A549 cells (*n* = 5) and treated with LSH for 14 days. (B) The tumors were removed from the tumor model mice, and their diameters were measured (*n* = 5). (C) Statistical analysis was conducted on the volume and weight of the tumors (*n* = 5). (D) The expression of Bcl‐2, Bax, caspase‐3, cleaved‐caspase‐3, PARP1, and cleaved‐PARP1 in the tumorous tissues was detected by Western blotting; the bar chart on the right shows the statistics of gray value scanning of protein bands (*n* = 3). (E) Immunohistochemical (IHC) staining was used to detect the expression of caspase‐3, Ki‐67, and Bcl‐2 in the tumorous tissues. The data in each group were expressed as mean ± SD, ^ns^ *p* > 0.05, ^∗^ *p* < 0.05, ^∗∗^ *p* < 0.01, ^∗∗∗∗^ *p* < 0.0001. The picture represents the results of three repeated experiments.

### 3.8. LSH Has No Obvious Side Effects and Toxic Effects on Normal Tissues and Organs

During the LSH treatment of NSCLC, the body weight of mice was measured every 4 days, and there was no significant difference in body weight between the LSH‐treated group and the untreated group (*p* > 0.05) (Figure 8A). To investigate the toxic effects of LSH on animal tumor models, we treated animals with low doses of LSH (LSH‐L) and high doses of LSH (LSH‐H). The signs of toxicity, such as loss of appetite and decreased activity or lethargy, were observed in each group of mice, and there was no significant difference between the LSH‐treated group and the untreated group. Liver function biomarkers, ALT and AST, and renal function biomarkers, CREA and BUN, in the untreated group and LSH‐H–treated group of mice were detected. The detection results showed that serum levels of ALT, AST, CREA, and BUN were not significantly different between the untreated group (*p* > 0.05) and the LSH‐H treated group, where the results of CREA and BUN are plotted (Figure 8B). Hematoxylin–eosin (HE) staining results of different organs (lungs, liver, heart, and kidney) in the untreated group and the LSH‐H–treated group showed that no significant histological damages were found in the lungs, liver, heart, and kidney in the LSH‐H–treated group compared with the treated group (Figure 8C). These results showed that LSH did not have significant toxicity in normal tissues and organs.

**FIGURE 8:**
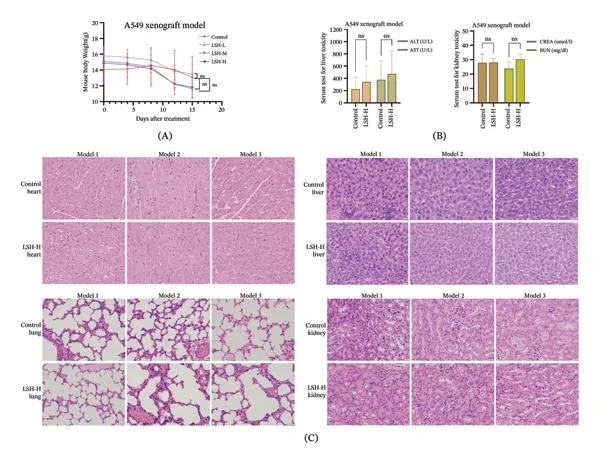
The cytotoxic effects of LSH on normal tissues and organs. (A) Body weight changes of mice in each group. There was no significant difference in body weight between the LSH treatment group and the control group (*n* = 5). (B) Serum renal function biomarkers (CREA and BUN) levels were not significantly different between the LSH‐H (high dose) group and the control group (*n* = 5). (C) HE staining of different organs (lungs, liver, heart, and kidney) in the LSH‐H group and control group showed that no significant histological differences were found in lungs, liver, heart, and kidney. The data in each group were expressed as mean ± SD. ^ns^ *p* > 0.05.

## 4. Discussion

According to Global Cancer Statistics 2020, lung cancer is the second most common cancer and the leading cause of cancer death in 2020, accounting for approximately one in 10 cancers diagnosed (11.4%) and one in five deaths (18.0%). Among men, lung cancer is the most common cancer in 36 countries, while it is the leading cause of cancer death in 93 countries [22]. According to the American Cancer Society in 2024, of the 125,070 lung cancer deaths, approximately 101,300 (81%) will be directly caused by smoking, and another 3500 will be caused by secondhand smoke. NSCLC is the most common type of lung cancer. Due to the lack of effective screening tools and early clinical symptoms, most NSCLC patients are diagnosed in the late stage, which seriously threatens the survival of patients. At present, the treatment of lung cancer includes surgery, radiotherapy, chemotherapy, and targeted therapy. In addition, new therapeutic strategies such as immunotherapy have been applied to the treatment of NSCLC, but the survival rate of NSCLC is still low due to treatment resistance [23]. It is of great significance to explore the prevention and therapeutics of NSCLC and develop effective drugs for the treatment of NSCLC.

There are 50 species of Stephania plants in the world, widely distributed in tropical and subtropical areas. According to the Flora of China, there are 39 species of Stephania in China. As a traditional Chinese herbal medicine, LSH can be used as medicine. It can be decocted and taken internally or mashed for external application. It has anti‐inflammatory and analgesic effects, but no studies have proved whether it has antitumor effects. Antitumor effects of other plants in the genus Stephania have been reported. For example, cepharanthine, a double benzyl isoquinoline alkaloid, was obtained from the money‐hanging gourd (*Stephania cepharantha* Hayata). These chemical components have significant antitumor activity on both p53 wild‐type and *P53* mutant colorectal cancer cells and are more effective in the growth control of *P53* mutant colorectal cancer cell lines HT‐29 and SW‐620 [24]. Cepharanthine has significant cytotoxic effects on chemotherapy‐sensitive CaOV‐3 and chemotherapy‐resistant OVCAR‐3 ovarian cancer cell lines, inhibiting the growth of CaOV‐3 and OVCAR‐3 cells in a time‐ and dose‐dependent manner [25]. Based on network pharmacology and molecular docking methods, this study initially explored the possibility and potential molecular mechanism of LSH anti‐NSCLC and proved the effect and molecular mechanism of LSH anti‐NSCLC through in vitro and in vivo experiments.

The UHPLC–OE–MS method was used to detect LSH components, and the top 20 active components were screened according to OB, DL, and MS2 score. The SwissTargetPrediction database predicted and screened 576 role molecular targets of LSH. A total of 2434 targets in NSCLC were obtained using GeneCard and OMIM databases; 261 overlapping targets were obtained by drawing a Venn diagram by Venny2.1.0. The PPL network was obtained by the STRING12.0 database, and 48 core targets were screened by Cytoscape software. Among them, the top 10 core targets are AKT1, STAT3, EGFR, HSP90AA1, SRC, GAPDH, JUN, BCL2, CASP3, and CCND1. Gene and protein expression levels of these four core targets are closely related to the occurrence and development of NSCLC and also affect the survival of patients with NSCLC. Next, GO and KEGG enrichment analysis was performed on the cross targets, and the “LSH‐active component–core target–pathway–NSCLC” network was constructed. The network topology analysis showed that the top 11 components associated with core targets were N‐trans‐feruloyltyramine, magnoflorine, genkwanin, acacetin, boldine, kaempferol 3‐methylether, aurantiamide, 7‐methoxybaicalein, (S)‐scoulerine, eriodictyol, and aurantiamideacetate. The genes most associated with both active components and pathways were EGFR, MAPK1, MAPK3, PIK3R1, PIK3CA, AKT1, IGF1R, MMP9, PTGS2, and SRC. Through GO and KEGG pathway enrichment and network topology analysis, it can be seen that the signaling pathway with the most core target correlation is pathways in cancer. The second were the PI3K/Akt signaling pathway, proteoglycans in cancer, the MAPK signaling pathway, and EGFR tyrosine kinase. This suggests that LSH has a therapeutic effect on NSCLC, which may mainly act on NSCLC through BP such as regulating protein phosphorylation by the PI3K/Akt signaling pathway, transmembrane receptor protein tyrosine kinase signaling pathway, and regulating tumor energy metabolism. The 11 key active components of LSH and their predicted core targets (EGFR, PIK3CA, PIK3R1, AKT1, HSP90AA1, SRC, MAPK1, MAPK3, JUN, and CASP3) were verified by molecular docking according to the CDOCKER method. Each of the key effective compounds and the predicted key core targets obtained a good docking effect of the complex, proving that LSH may play an anti‐NSCLC role through these key core targets. Literature analysis indicates that by using network pharmacology and molecular docking methods to observe the target sites of the traditional Chinese drug kaempferol in the treatment of NSCLC, PIK3R1, AKT1, EGFR, and IGF1R are likely to be the key targets [26]. Our study also indicates that the target of LSH involves the inhibition of the PI3K/AKT signaling pathway, implying that the PI3K/AKT signaling pathway is a key target for these traditional Chinese drugs in the treatment of NSCLC.

To mitigate the potential impact of cellular senescence during cell passages and individual variability among animals on the experimental results, this study incorporates not only technical replicates but also biological replicates (across different subcultures or independent animal groups). We established stringent control groups to prevent any instability in the results arising from such variability. This study verified that LSH could inhibit the proliferation, migration, and invasion of lung cancer A549 cells in a dose‐dependent manner through the CCK‐8 assay, plate cloning assay, EdU cell proliferation assay, scratch assay, and invasion assay, and verified that LSH promoted the death of A549 cells through microscopy and X‐ray electron microscopy. Flow cytometry and Western blotting analysis proved that LSH induced apoptosis of A549 cells. The mouse xenograft A549 model was treated by LSH, compared with the untreated group. In the treatment group, tumor volume and weight decreased, and the expression of Ki‐67, caspase‐3, cleaved‐caspase‐3, and Bax/Bcl‐2 was significantly increased by IHC staining and Western blotting detection. The dose‐dependent anti‐NSCLC effect of LSH was demonstrated. The results of CCK‐8 experiments on normal human lung MRC‐5 cells showed that LSH had selective cytotoxic effects on NSCLC cells, while the toxicity to normal lung cells was very low. After the end of the treatment period in animal experiments, serum liver and kidney toxicity marker analysis and HE staining results of different organs (lung, heart, liver, and kidney) showed no significant difference between the untreated group and the LSH‐H–treated group. These results indicated that LSH is less toxic to various organs in the body and has the feasibility of becoming a clinical drug.

The epidermogenic factor receptor (EGFR) is a receptor tyrosine kinase (RTK) belonging to the ErbB family, whose expression is regulated by specific transcription factors. Inhibition of the EGFR/PI3K/AKT signaling pathway can inhibit proliferation, invasion, and migration of NSCLC cells [27]. In the present study, the results indicated that LSH was able to inhibit the expression of p‐AKT (Ser473) in lung cancer cells, suggesting that LSH may act on the PI3K/AKT signaling pathway by targeting EGFR, STAT3, PIK3CA, PIK3R1, AKT1, HSP90AA1, and IGF1R, inhibit the proliferation, migration, and invasion of NSCLC cells, and promote apoptosis, thereby being able to treat NSCLC. In tumor cells, regardless of oxygen content, pyruvate in tumor cells is mainly converted into lactic acid to produce ATP, thus maintaining sufficient macromolecular synthesis substrate for the proliferation of active tumor cells, a phenomenon known as the Warburg effect [28]. The underlying mechanism of the Warburg effect is complex, involving activation of oncogenes, inactivation of tumor suppressor genes, abnormal expression of glycolytic enzymes, and changes in the tumor microenvironment [29]. Regulation of glycolytic products is a part of metabolic reprogramming and is closely related to the genesis and development of tumors. The PI3K/AKT/mTOR pathway is one of the most critical regulatory pathways of the Warburg effect in malignant tumor cells [30]. Combined with multiomics and network pharmacology, it is speculated that fuzialkaloids may regulate glycolysis through the PI3K/AKT/mTOR signaling pathway, thereby inhibiting the growth of NSCLC cells [31]. Through network pharmacology and GO and KEGG pathway analysis, this study has found many core targets associated with proteoglycans in cancer, suggesting that LSH may regulate glycolysis through the PI3K/AKT/mTOR signaling pathway to inhibit the growth of NSCLC cells. Growth faction‐induced MAPK pathway signal transduction plays an important role in PD‐L1 expression in lung adenocarcinoma. EGFR and MEK1/2 inhibitors can both decrease EGF and IFNγ‐induced PD‐L1 expression, which may increase the immunogenicity of lung adenocarcinoma cells. However, because the MAPK pathway lies downstream of a large number of growth factor receptors, downstream inhibition using MEK1/2 inhibitors may regulate PD‐L1 more effectively than inhibition of specific growth factor receptors. It has been found that MEK1/2 inhibition (but not EGFR inhibition) prevents HGF‐induced PD‐L1 expression [32]. In the present study, the results of network pharmacology and molecular docking indicated that LSH may act on the MAPK pathway by targeting MAPK1 and MAPK3 to anti‐NSCLC. Src is a nonreceptor protein tyrosine kinase involved in regulating gene transcription, immune response, cell adhesion, cell cycle progression, apoptosis, and migration signaling pathways, and is an important therapeutic target. Administration of Src or ceramidase inhibitors has been shown to salvage the response of chemotherapy‐resistant pancreatic and lung cancer cells to chemotherapy by reactivating pyrodeath in vitro and in vivo [33]. Bcl‐2 family members are key regulators of cell death or survival, and its member Bax is a major proapoptotic protein whose activation is necessary for cell death. Under the action of growth factors, protein kinases such as AKT mediate the phosphorylation of Bax, activate its proapoptotic activity, and induce NSCLC cell longevity [34]. Different protease cleavage activates caspase‐3 to become cleaved caspase‐3, which further cuts different substrates, leading to protease cascade amplification and ultimately cell death [35]. The cleaved substrate leads to altered protein function, which leads to cellular changes associated with apoptosis, which is induced by caspase‐3 activation [36]. The results of this study showed that after LSH treatment of lung cancer cells, the expression levels of caspase‐3 and cleaved caspase‐3 were increased, and the ratio of Bax/Bcl‐2 expression levels was also increased, suggesting that LSH could promote apoptosis of NSCLC cells. Recent studies have highlighted pyroptosis as a form of inflammation with programmed cell death as a potential strategy to overcome drug resistance. Researchers are studying the relationship between pyroptosis and apoptosis to advance cancer therapy [37]. In recent years, caspase‐3/GSDMe–dependent pyroptosis signaling pathways have been discovered. Downstream of activated caspase‐3, GSDME forms an N‐terminal domain that recognizes and punctures cell membrane perforation, leading to cell expansion and rupture and the release of inflammatory factors and DAMage‐related molecular patterns (DAMPS) [38]. In this study, the results showed that treatment of A549 cells with LSH may promote pyroptosis. TEM revealed membrane blistering and signs of cell death, indicating that LSH may inhibit the growth of NSCLC cells by inducing apoptosis or ferroptosis in these cells.

There are more active components in LSH and more molecular targets and signaling pathways acting on NSCLC. Different components may have the same target; that is, the same molecular target can become the target of multiple components [39]. LSH may promote apoptosis or ferroptosis of NSCLC cells and inhibit the proliferation, migration, and invasion of cancer cells. LSH plays a role in the treatment of NSCLC, maybe through multicomponent targeting of multimolecules and multisignaling pathways, and emerges as a good effect in the treatment of NSCLC.

## 5. Conclusion

This study found that LSH can inhibit the growth of NSCLC both in vitro and in vivo. LSH can exert anti‐NSCLC effects by multicomponent targeting multimolecule and multisignaling pathways. LSH inhibits EGFR, AKT1, STAT3, HSP90AA1, and SRC, which may be the key target molecules in anti‐NSCLC. The findings of this study indicate that LSH exhibits anti‐NSCLC efficacy while demonstrating no significant toxic effects on normal cells or organs. These results support the initiation of further preclinical studies, thereby establishing a preliminary experimental foundation for the development of novel herbal medicine for treating cancer.

**Nomenclature**
LSH: Long stephania herb
NSCLC: Non‐small cell lung cancer
EGFR: Epidermal growth factor receptor
TKIs: Tyrosine kinase inhibitors
TCM: Traditional Chinese medicine
BXD: Banxia Xiexin decoction
PPI: Protein–protein interaction
GO: Gene Ontology
CC: Cellular component
KEGG: Kyoto Encyclopedia of Genes and Genomes
BP: Biological processes
PDB: Protein Data Bank
MF: Molecular functions
ALT: Alanine aminotransferase
AST: Aspartate aminotransferase
CREA: Creatinine
BUN: Blood urea nitrogen
HE: Hematoxylin–eosin
CCK‐8: Cell Counting Kit‐8
VEGFR: Vascular endothelial growth factor receptor
TKI: Tyrosine kinase inhibitor
PI3K: phosphatidylinositol 3‐kinase
AKT: Protein kinases
AKT1: AKT Serine/threonine kinase 1
STAT3: Signal transducer and activator of transcription 3
HSP90AA1: Heat shock protein 90 alpha family class A member 1
SRC: SRC proto‐oncogene, nonreceptor tyrosine kinase
GAPDH: Glyceraldehyde‐3‐phosphate dehydrogenase
JUN: Jun proto‐oncogene, AP‐1 transcription factor subunit
BCL2: B‐cell lymphoma 2
PIK3CA: Phosphatidylinositol‐4,5‐bisphosphate 3‐kinase catalytic subunit alpha
PIK3R1: Phosphoinositide‐3‐kinase regulatory subunit 1
MAPK1: Mitogen‐activated protein kinase 1
MAPK3: Mitogen‐activated protein kinase 3
BPDGFR‐β: Beta platelet‐derived growth factor receptor
FBS: Fetal bovine serum
SDS: Sodium dodecyl sulfate
PBS: Phosphate‐buffered saline
IHC: Immunohistochemistry
OB: Oral bioavailability
DL: Drug‐like properties
TCMSP: Traditional Chinese Medicine Systems Pharmacology Database and Analysis Platform
OMIM: Online Mendelian Inheritance in Man
RCSBPDB: Research Collaboratory for Structural Bioinformatics Protein Data Bank
PubChem: Public Chemical Database
STRING: Search Tool for the Retrieval of Interacting Genes/Proteins

## Author Contributions

Xiaobing Chen, Luying Zhang and Wanying Zhang participated in the experimental design and execution, contributed to key experimental data and analysis, and drafted the initial manuscript. Xueqin Wu, Kun Liu, Yuli Zhou, and Yongqing Chen were involved in part of the experimental operations and data analysis. Mingyue Zhu participates in the discussion and analysis of research data. Bo Lin and Mengsen Li, as project leaders, co‐guided the entire research and participated in the experimental design and discussion of the results and wrote the final revision of the manuscript.

## Funding

This work was supported by the National Natural Science Foundation of China (Nos. 82460602, 82573045, 82560459, 81960519, and 82060514); the Hainan Province Science and Technology Special Foundation (No. ZDYF2021SHFZ222); the Natural Science Foundation of Hainan Province (No. 824RC517); and the Hainan Provincial Graduate Innovation Research Project (No. Qhys2023‐450).

## Disclosure

All authors read and approved the final manuscript.

## Conflicts of Interest

The authors declare no conflicts of interest.

## Supporting Information

Additional supporting information can be found online in the Supporting Information section.

## Supporting information


**Supporting Information** More than 1000 compounds were identified from the LSH water extracted medicinal liquid sample, the specific information of which included MS2_name, formula, pubchemid, MS2_score, and others, and the full information of all substances detected by LSH.

## Data Availability

The original data generated in this study are included in the article. If necessary, the corresponding author can be contacted directly.
